# Supplementary material for: Splice-Junction-Based Mapping of Alternative Isoforms in the Human Proteome
Source: Cell Rep. Author manuscript; Available in PMC 2020 Jan 15. (PMC6961840; doi:10.1016/j.celrep.2019.11.026)
Supplement: 2 [file NIHMS1546469-supplement-2.zip › DF1/PXD010154/Pancreas/Pancreas_3_NA_AGAQAAGSTWVLHETFSQH.pdf]

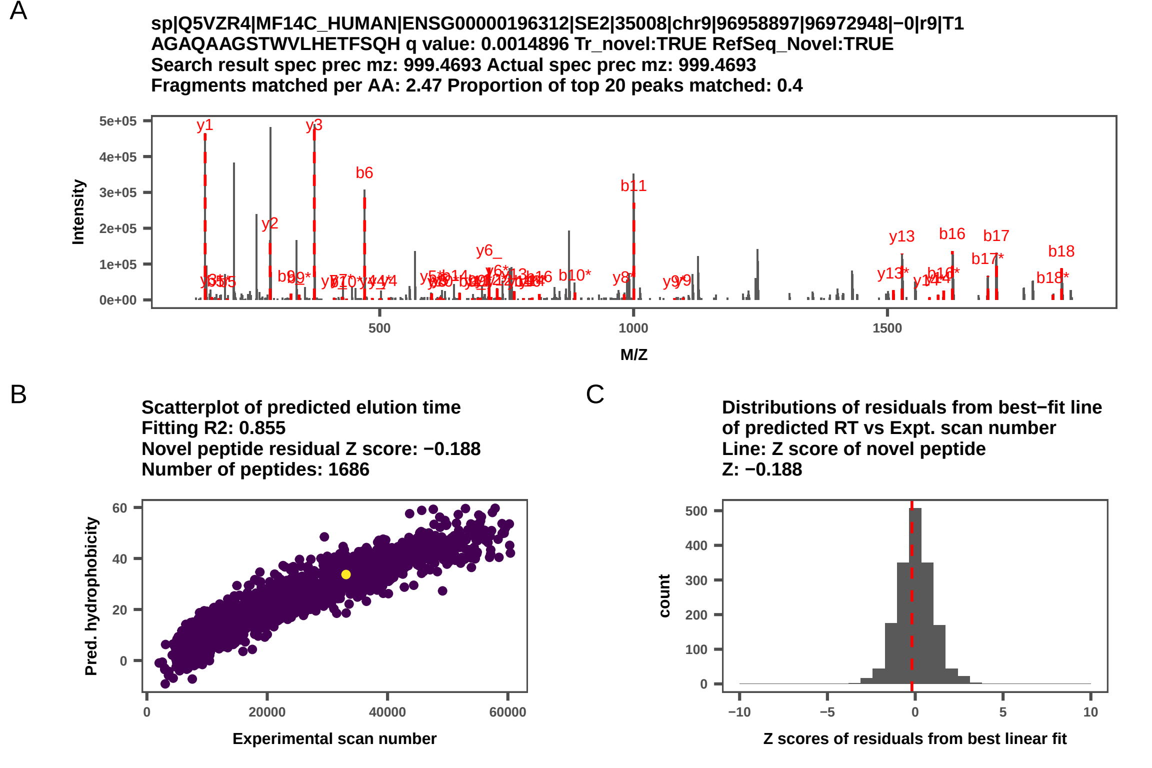
A
sp|Q5VZR4|MF14C_HUMAN|ENSG00000196312|SE2|35008|chr9|96958897|96972948|−0|r9|T1
AGAQAAGSTWVLHETFSQH q value: 0.0014896 Tr_novel:TRUE RefSeq_Novel:TRUE
Search result spec prec mz: 999.4693 Actual spec prec mz: 999.4693
Fragments matched per AA: 2.47 Proportion of top 20 peaks matched: 0.4
Intensity
5e+05
4e+05
3e+05
2e+05
1e+05
0e+00
y1
y3
b6
y2
b11
y13
b16
b17
b17*
b18
b18*
b10*
y13*
b16*
M/Z
500
1000
1500
B
Scatterplot of predicted elution time
Fitting R2: 0.855
Novel peptide residual Z score: −0.188
Number of peptides: 1686
Pred. hydrophobicity
60
40
20
0
0
20000
40000
60000
Experimental scan number
C
Distributions of residuals from best−fit line
of predicted RT vs Expt. scan number
Line: Z score of novel peptide
Z: −0.188
count
500
400
300
200
100
0
−10
−5
0
5
10
Z scores of residuals from best linear fit
